# Work Environment-Related Factors in Obtaining and Maintaining Work in a Competitive Employment Setting for Employees with Intellectual Disabilities: A Systematic Review

**DOI:** 10.1007/s10926-015-9586-1

**Published:** 2015-06-26

**Authors:** Joke J. H. Ellenkamp, Evelien P. M. Brouwers, Petri J. C. M. Embregts, Margot C. W. Joosen, Jaap van Weeghel

**Affiliations:** University Tilburg, Tilburg, The Netherlands

**Keywords:** Intellectual disability, Developmental disability, Paid work, Competitive employment, Labor participation

## Abstract

*Background* People with an intellectual disability value work as a significant part of their lives, and many of them want to participate in regular paid employment. Current estimates show that the number of people with ID who have some form of paid employment are very low, ranging from 9 to 40 % across different countries, despite legislations. This review examines papers published in the past 20 years in an attempt to answer the following research question: ‘What work environment-related factors contribute to obtaining or maintaining work in competitive employment for people with an intellectual disability?’ *Method* The databases of PubMed, PsycINFO, CINAHL, Embase and Web of Science were searched for relevant papers published between 1993 and 2013. All papers were independently screened by two researchers. Methodological quality of the studies was evaluated, and data on work environment-related factors stimulating employment for people with intellectual disabilities were extracted and grouped into categories. *Results* A total of 1932 articles were retrieved. After extensive screening for relevance and quality, 26 articles were included in this review. Four themes/categories with work environment-related factors that could influence work participation were distinguished. Five studies were conducted on employers’ decisions and opinions. Eight focused on job content and performance, and eight on workplace interaction and culture. Five studies evaluated support by job coaches. *Conclusion* Despite ongoing legislation to promote participation of people with intellectual disabilities in the paid workforce, research in this area is still extremely scarce. In the past 20 years, very few studies have focused on work environment-related factors that can enhance competitive work for people with intellectual disabilities. This review shows that relevant work environment-related factors for obtaining and maintaining work in competitive employment include supporting the employers by paying specific attention to: employer’s decisions, job content, integration and work culture and job coaches.

**Electronic supplementary material:**

The online version of this article (doi:10.1007/s10926-015-9586-1) contains supplementary material, which is available to authorized users.

## Introduction

People with an intellectual disability (ID) indicate that working is a significant part of their lives, and many of them would want to participate in regular paid employment [1]. Having a regular job offers a range of benefits for example conveying status and purpose to an individual‘s existence, promoting participation and social interaction with others in society. It is also an opportunity for financial autonomy and a social determinant of health in that it promotes quality of life, better health, and a greater sense of control over one’s life [1–3]. In addition, work allows to learn new skills and can contribute to a higher self-esteem [4–8]. Estimates of the prevalence of people with ID vary between three and six persons per 1000 and depend on diagnostic criteria, severity of disability, study population and socio-economic status [9].

Historically, people with ID have not had access to regular paid employment; most vocational services were provided in segregated workplaces [10]. Growing attention of the human rights movement to normalization, integration and deinstitutionalization led to a change in attitude about work for people with ID, and to new legislation and policies [11, 12]. Inclusion of people with ID in the workforce was recently emphasized by the United Nations in the ‘Convention of human rights for people with disabilities’ (2006). Despite legislation and policy documents, there is still a considerable gap between the employment rates of people with and without disabilities [13–17]. Current estimates of the number of people with ID who have some form of paid employment are very low, reported to range from 9 to 40 % across different countries [1, 10, 17–21].

Supported employment is an evidence based practice and defined as employment for people with disabilities in the competitive labor market working in jobs they prefer for real pay with the level of professional help that is needed [22]. However, very few studies have focused on work environment-related factors that influence work participation of persons with an ID. Most studies examined individual factors and school programs. The importance of motivation, competence, self-determination and working behavior as examples of individual factors was stressed in many studies [1, 18, 20, 23–27]. Studies focusing on school programs described the importance of a smooth transition from school to work, as well as support at the workplace [28–30].

Beside individual factors and school programs the context or environment is important in understanding the individual functioning of people with ID. Some studies investigated social inclusion, natural supports and participation in relation to community-based employment [12, 31, 32]. One review study was based on social inclusion as an outcome of employment settings [33]. Eleven components of social inclusion were distinguished in this study of which social roles, personal skills and characteristics and the environment were most frequently mentioned. The well-known International Classification of Functioning, Disability and Health (the ICF model) and the later World Report on Disability, identify both individual and environmental factors that are important in understanding individual functioning [34, 35]. The World Report on Disability suggests steps to be taken by stakeholders to create environments that are beneficial to people with a disability including ID, including increasing public awareness and understanding and the adoption of national strategy plans.

The present review focuses on work environment-related factors and defined it as factors that can be influenced by the employers and have to do with the work setting. Legislation or policy documents are not included or discussed here. This review addresses the question: ‘What work environment-related factors contribute to obtaining or maintaining work in competitive employment for employees with ID? This knowledge will hopefully provide input for employers that already do, or would like to work with individuals with ID. This information is additional to the results of earlier studies focusing on individually-related factors or on school programs.

## Method

### Search Strategy

A systematic search was made in the databases of Pubmed, PsycINFO, Web of Science, Embase and CINAHL for articles published in English between January 1993 and April 2013. This period is chosen because supported employment started in the 1980s in the USA from where it spread to Canada and Western countries and it was decided not to use studies older than 20 years.

The search terms used for the databases were divided into two domains (ESM Appendix 1). The first concerned the population under study, i.e. persons with developmental-, learning- or intellectual disabilities (and their synonyms). The second domain concerned the outcome for research on paid work, competitive employment, labor and supported employment (and their synonyms); all these terms were modified for each database as required (ESM Appendix 2). No constraints were made related to the level of an individual’s IQ.

### Study Selection

Selection of the articles was divided into four consecutive phases.

The *first* phase was based on the title of the article; for this phase inclusion criteria were: (1) people with ID based on the criteria of the American Association of Developmental Disabilities (AAIDD) and (2) with paid work in a regular setting. In the *second* phase abstracts were rated using the inclusion criteria used in the first phase supplemented with: (3) empirical data as basis for information on different work settings and (4) work environment-related factors that could be influenced by employers. Excluded were articles which reported mixed populations in which results for persons with ID were not reported separately (e.g. where studies in which subjects were people with ID and/or psychiatric patients). Other exclusion criteria were: lack of clarity about the definition of ID, no paid work or working <4 h a day, no relation with work environment-related factors, editorial/review, articles on policy and legislation. The *third* selection phase consisted of rating the full text articles and the *fourth* phase comprised quality assessment based on the full text.

**Fig. 1:**
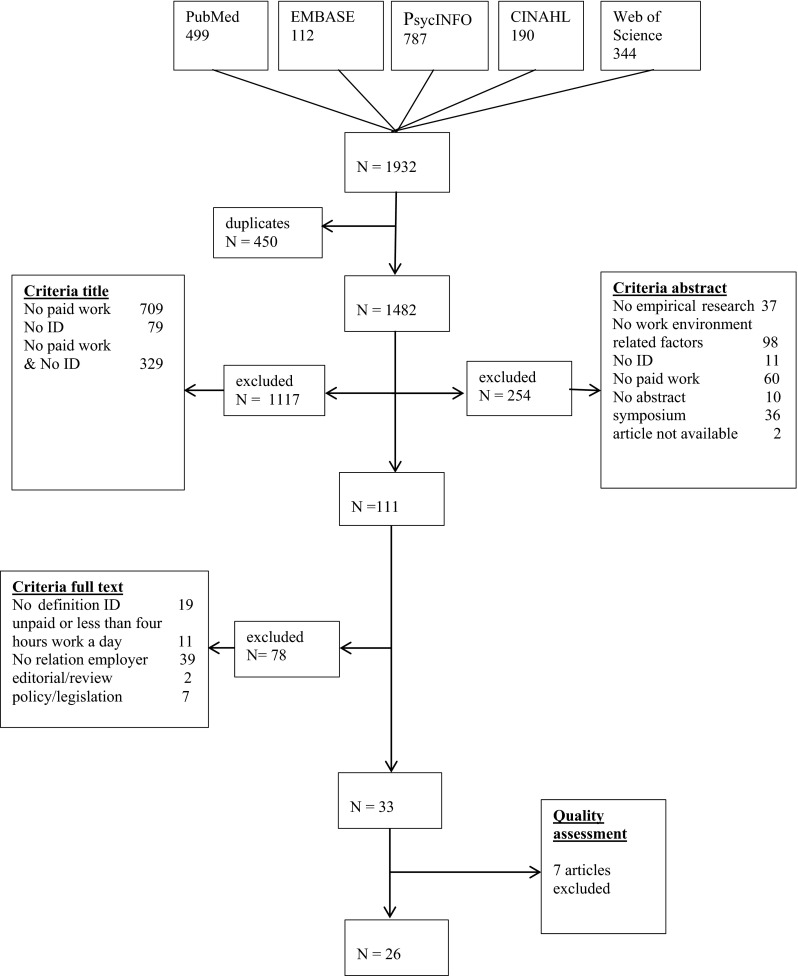
Flowchart

All four phases were conducted by two authors who independently reviewed the articles on inclusion/exclusion criteria in all stages of the selection process. One author (JE) read all the selected titles, abstracts and articles, and collected the outcomes from the other authors after each step. All other co-authors (JvW, EB, PE/MJ) read one-third of the materials each. If the authors made different choices in the different phases, these were discussed until consensus was reached. In case of remaining doubt about inclusion in any of these steps, the articles were included in the next step of the selection process. Figure 1 is a flow chart of the selection process.

### Quality Assessment, Data Selection and Data Analysis

In the fourth phase, all articles were assessed on methodological quality using eleven quality indicators developed by Buckley et al. [36]. This quality instrument was used because it is applicable to both qualitative and quantitative studies. The indicators were related to: the appropriateness of the study design, study conduct, analysis of results, and the conclusions (Table 1). Higher quality studies were considered to be those studies with a score of at least seven of these eleven indicators as defined by Buckley et al. [36].

**Table 1:** Quality indicators

Quality indicators for included studies based on Buckley et al. [36]	
Research question	Is the research question(s) or hypothesis cleary stated?
Study subjects	Is the subject group appropriate for the study being carried out?
‘Data’ collection methods	Are the methods used reliable and valid for the research question and context?
Completeness of ‘data’	Have subjects dropped out? Is the attrition rate les than 50 %?
Control for confounding	Have multiple factors/variables been removed or accounted for where possible?
Analysis of the results	Are the statistical or other methods of result analysis used appropriate?
Conclusions	Is it clear that the data justify the conclusions drawn?
Reproducibility	Could the study be repeated by other researchers?
Prospective	Does the study look forwards in the time rather than backwards?
Ethical issues	Were all relevant ethical issues addressed?
Triangulation	Were results supported by data from more than one source?

This quality instrument was used as a screenings instrument in the last phase. One item of the eleven items of this screenings instrument was not applicable for all studies concerning control of confounding. Therefore we used a cut-off of six instead of seven of the eleven criteria for the assessment of high quality of the included studies as defined by Buckley et al. [36].

From the selected articles four categories of themes emerged. Subsequently, all papers were grouped into these categories (see Table 2). From the selected articles the information on the following items was extracted: author(s), year of publication, country, study population, sample size, type of work settings, primary focus of study, research design and outcome measures.

**Table 2:** Data extraction table of included articles

Country and author	Primary focus of study	Study population and sample size	Type of work settings	Outcome measures	Results	Research design
*Employers’ decisions and opinions (N* *=* *5)*						
Canada Blessing [41]	Employers attitudes and selection decisions in hiring people with ID	20 employers with experience with people with ID and 18 employers without such experience N = 38	Manufacturing and technical services, food services; laundry and dry cleaning; retail and other services and clerical work	Assessment of employers attitudes towards employees with ID, identifying employer perceptions of barriers, incentives and knowledge in what way past experience influences the selection process	Injury was most cited as barrier to employment of this population. Incentive worksite factors were social altruism, personal satisfaction and growth, assistance in repetitive tasks, additional support systems and individualized expectations	Questionnaire and interviews with employers
USA Morgan and Alexander [40]	Employers with/without experience in hiring employees with ID	Employers N = 534	Service; manufacturing; retail; technical; building; medical; financial; professional; education; transportation; farming; government and other	Nature of business, hiring experience of workers with ID, advantages and concerns about employing individuals with ID and future hiring intent	Less than one-third of respondents reported experience in hiring persons with disabilities. Most employers with experience would hire again in the future. Loss of productivity and unsafety were relevant barriers for employing persons with ID	Questionnaire filled in by employers
New Zealand Reid [42]	Multiple perspectives in realizing greater employment rates (workers with ID N = 17; employers N = 3; Support people N = 7; experts N = 2)	Financial independent employees with ID N = 29	Community social/personal service sector (N = 10); construction sector (N = 2); manufacturing sector (N = 2), transport sector (N = 2), wholesale. Retail and hotel/restaurant sector (N = 1)	Support of workers, getting the job, learning on the job and maintaining the job	Support of another person and formal social support at the work place was important to get and maintain a job. Workers with ID were highly motivated and identified other and more work than they would like to do	Interviews with employers
Hong Kong Tse [38]	Employers’ decision making processes	Employers hiring employees with ID N = 66	Manufacturing industries (N = 42); service industries (N = 13); commercial sector (N = 7); retail (N = 2); public sector (N = 2)	Twenty-two items as composed who examined employers’ attitudes towards persons with disabilities	Most important factors affecting employers’ decisions were: emotional problems and personalities of workers, workers’ ability to perform a job, availability of low-level jobs, productivity of workers and availability of supportive services	Telephone interviews and a questionnaire for employers
Canada Wilgosh et al. [39]	Experiences of employers with workers with ID	Employers who hire employees with ID in entry level positions N = 41	Food service, janitorial/housekeeping, retail and labor/assembly	Thirteen skills were identified based on a questionnaire and how they influenced the decision process of employers to hire employees with ID	Most important skills for employers were attendance, production and work attitude. Additional prevocational preparation and training for job readiness and on the job training were important for decisions concerning placement and keeping the job	Interviews with employers
*Job content, requirement and performance (N* *=* *8)*						
USA Browder and Minarovic [45]	Training package of sight words to initiate job tasks	Employees with moderate or mild ID N = 3	Grocery store, cafeteria, clothing factory	1. Correct number of sight words read; 2. Know what to do after sight word reading; 3. Initiations undertaken by employees for performing tasks; 4. Satisfaction of employer	Training was effective for all participants to initiate tasks in their work setting. The used method supported them in self-management on the job and employers’ ratings of the job performance was positive	Experimental design with observations
USA Devlin [43]	Impact of self determined career development model on job performance	Employees with moderate ID N = 4	Cleaning duties	Self-determined career development model (SDCDM) about self-selected goals and how they were reached	The SDCDM represented an effective method to teach problem solving and suggests a positive relationship between the use of the model and changes in job performance	Experimental design with observations
Japan Ishii and Yaeda [46]	Performed tasks by employees with ID and differences between companies	Employers N = 150	Task descriptions were made of most frequently performed tasks	1. Tasks performed by employees; 2. Job development activities used as predictive activities to clarify the prevalence of these activities; 3. Job development activities	Clerical, custodial and manufacturing work was most frequently performed. Companies employing individuals with ID provided significantly more job development activities than those not hiring such individuals. Job coach needed to offer employers professional consultations helping to develop jobs	Questionnaire filled out by employers
Spain Flores [48]	Quality of working life	Employees with ID in supported employment N = 65	29 % concierge in an office and 12.9 % attendance in a shop, other work settings not mentioned	Job demands (job, task and psychological and physical) and resources (organizational, interpersonal, social supports from co-workers and supervisor and quality life)	Reduced job demands, elevated support from supervisor and co-workers explained quality of working life	Cross-sectional survey with interviews with employees
Hong Kong Li [49]	Self-perceived opportunities for getting a job	Employees with mild disability (IQ 50–70) N = 18	Cleaning work (N = 9), security guard (N = 1), factory worker (N = 2), messenger (N = 3), store assistant (N = 1), sheltered workshop (N = 1), unemployed (N = 1)	Self perceived employment opportunities for people with ID with average or above average work abilities	Employees were worried about work prospects and confronted with misconceptions. Suggestions were made for promoting work by professional assistance, disability education for employers, training to improve work performance and self-advocacy of people with ID	Interviews with employees
USA Melchiori and Church [44]	Self reported vocational needs and satisfaction of supported employees and co-workers	Supported employees with ID and their co-workers N = 45	Manufacturing or product assembly (N = 15); janitorial services (N = 9); recycling (N = 6); food service (N = 5); retail customer service (N = 4); office work (N = 4); research support (N = 1); child care (N = 1)	Instruments based on the theory of work adjustment in which person-environment correspondence predicts worker adjustment from interaction between workers’ personalities and work environment to identify vocational needs and satisfaction of workers with ID and their co-workers	Supported employees were more satisfied than co-workers with the variety of their work tasks, supervision, compensation, working conditions, recognition and ability utilization. Theory of Work Adjustment could be used in clarifying the vocational needs and facilitate active participation in vocational decision making	Questionnaire filled in by supervisors
USA Pierce et al. [47]	Predictors of job tenure	Employees with ID N = 317	Top five employment: 1. Food service; 2. Grocery; 3. Disability board; 4. Retail; 5. Motel	Employment status 1998, 3 year employment status, movement to a competitive job	Matching interest and abilities reduced problems in job retention. Increase in wages and hours was a relevant factor for remaining in a job for more than a year	Prospective cohort study over 3 year period using local county providers database
USA Rusch et al. [60]	Relation IQ, ethnicity, gender, placement approach and current/future earnings	Employees with ID (mean IQ 59.2) placed in supported employment N = 197	No information about job types only about monthly earnings	Intelligence, etnicity, job placement, job type, means of transportation, monthly earnings in relation to level of ID and job subsidy	Earnings were greatly influenced by keeping one’s job. Individual placed persons earn more than workers in enclaves. IQ and job subsidy were significant predictors for earnings	Prospective cohort study over 4 year period with random sample of state database
*Interaction and workplace culture (N* *=* *8)*						
USA Butterworth et al. [50]	Workplace culture	Young employees with ID working in individual community based jobs N = 8	Retirement home; rural and urban fast food restaurant, recycling center; funeral home; hair salon; convenience store; family style restaurant	1. Job Scale of the vocational inventory measuring how the environment responds to the needs of employees; 2. Consumer Scale of the vocational inventory measuring how the employee interacts with others	Four work setting characteristics were associated with a strong workplace culture: (1) multiple context relationships, (2) specific social opportunities, (3) personnel (4) teambuilding management style and job designs	Participant observation interviews with employees
USA Carrier [51]	Social integration	Employees with moderate, mild and severe ID N = 10	Rural-agricultural (N = 1), suburban- commercial (N = 2), urban commercial (N = 4), urban-industrial (N = 3)	Theory of co adaptation used for the workplace as a whole in which the expectations of the person with ID and motivations of employer are important	Three iterative transformation orders in a co-adaptation process were important to achieve optimal social integration; tasks adjustment, situational assessment, job creation and supervision	Participant observation and interviews with all stakeholders; questionnaire for job coaches
USA Chadsey Rusch et al. [60]	Interaction outcomes seen by different stakeholders	Employees with ID (N = 47), job coaches (N = 30), employers (N = 49) N = 126	50 % of employees worked in integrated work settings in offices or restaurants. The other work settings were not described	Social participation, workplace acceptance, personal acceptance, feelings of social support outcome, individual interventions and coworker/employer interventions	Time, money and staff were relevant factors for social integration by all the stakeholders. Workers with ID and job coaches believed that the barrier for social integration was problems in learning new skills as opposed to employers	Cross sectional survey with questionnaire for job coaches and employers and interviews for employees
USA Hall and Kramer [52]	Types of social networks and workplace structures in sheltered and community settings	Employees without explanation of level of ID in community employment N = 9	Pharmacy (N = 1); wholesale club (N = 1); discount department (N = 1), store (N = 1); camera store (N = 1); video store (N = 1); private school (N = 1); home goods store (N = 1), YMCA (N = 1)	Social capital defined as relationships and the benefits derived from these relationships in combination with external variables (organizational characteristics, living situation, family involvement, personality characteristics, perceived level of disability)	Commitment and leaderships is distinctive in creating social capital in workplaces with support of coworkers and supervisors. Contact with families was important and social capital produced bridges with people, places and ideas that otherwise would not be accessible	Interviews with employees
USA Irvine and Lupart [5]	Inclusion in the workplace	Employers N = 10	Work settings ranged from community businesses to charity organizations	Logistics, supports and accommodations, employee characteristics, perspectives of employee inclusion and advantages of the workplace	Benefits for the workplace by working with persons with ID were moral in the work environment, new role models and increased efficiency. Collaboration with supervisors, support staff and guardians was essential for workplace inclusion even as flexibility of the workplace	Interviews with employers
Australia Knox and Parmenter [54]	Social networks and support of these networks	Employees with mild ID N = 9	Bakery, trolley collector (N = 2) kitchen, factory (N = 2), ward assistant (N = 1), cleaner (N = 1), mail courier (N = 1), canteen assistant (N = 1)	Support defined in the categories: companionship; emotional and instrumental	Multiple support mechanisms were relevant for the workplace such as employers, co-workers and job coaches with special attention for the family	Participant observation over an 18-month period
USA Rusch et al. [53]	Co- workers interactions between employees with and without disabilities	Pairs of employees with and without ID in same jobs with mean IQ ranged from 52 to 59 in clustered or individual employment N = 85	Food service (N = 39), light industry (N = 16), service occupations (N = 18), other (N = 12)	1. Physical integration; 2. Social integration; 3. Training; 4/5 Associating (in frequency and appropriateness); 6. Befriending; 7. Advocating; 8. Evaluating; 9. Given information and 10. Individual and clustered placement	Employees with and without disabilities were interactive with their co-workers at similar levels in eight of the nine areas of interaction. The differences could be found in the different types of jobs. Significantly more off-the-job interaction occurs among workers without disabilities	Experimental design with matched pairs with use of a questionnaire
USA Rusch et al. [31]	Integration related issues emerging from co-worker interactions	Pairs of workers with severe ID in clustered or individual employment N = 23	Food industry (55 %); light industry (27 %); other placements (18 %)	See above mentioned elements with three different placement approaches	Employees without disabilities received more training and information from their co-workers without disabilities and nondisabled workers interact more as friends outside the workplace than supported employees	Experimental design with matched pairs
*Job coaches (N* *=* *5)*						
USA Gray et al. [55]	The effect of the number of job coaches on employment rates	Employees with ID with IQ (20–74) in competitive or enclave work settings N = 431	No information about work settings, only differences between type of urbanization	1. Job coaches per 100 individuals; 2. Percent urbanization; 3. Per capital unemployment rate; 4. Specific characteristics of employees with ID	Job coaches were successful in obtaining employment for individuals with IQ less than 40 and their effect on individual employment was positive and significant in highly urban counties	File analysis on data of 1996/1997 of the department of disabilities and special needs
USA Lemaire [70]	Employer reported barriers by job coaches to continued employment	Employees with mild to moderate ID who were eligible to receive services of developmental disabilities administration. N = 149	Laborer (36 %) food service (20 %), stocking, delivery (17 %) clerical worker (14 %), grocery store (6 %), service representative (4 %), parking assistant (3 %), other	Barriers reported by job coaches and reasons for employment termination	123 barriers were indentified for 49 employees and divided in behavior problems reading difficulties, legal history, transportation problems problems with supervisor and other	File analysis on records of supported employment services
USA Moore et al. [64]	Relation of gender, race, secondary psychiatric disability, type of training and transportation to work	Employees with mild or moderate ID N = 253	Nothing mentioned about the type of work setting only services delivered	Work status, level of income, transportation, vocational services, on –the-job training, maintenance, adjustment training	Job placement as a service was positively associated with achieving competitive jobs though not with the level of consumer income	File analysis on national database
USA Moore et al. [56]	Participants who were more likely to get competitive jobs and the role of support services	Employees with mild (60 %), moderate (34 %) and severe (6 %)mental retardation N = 28.565	No specific information	Level of IQ and vocational rehabilitation services (businesses and vocational training, counseling and job placement)	Consumers with a mild mental retardation who received job placement services achieved competitive jobs at a significantly higher proportion than persons with moderate or severe ID	File analysis on data provided by the rehabilitation services administration
USA Ward et al. [58]	Satisfaction of employers about the job coaches	Employers and job coaches with employees with ID N = 40	Fast food (N = 9), grocery (N = 6), retail (N = 3), bakery (N = 1), library (N = 2), restaurant (N = 1), animals (N = 2), laundry (N = 1), warehouse (N = 1), custodial (N = 5), child care (N = 2), newspaper (N = 1), housekeeping (N = 1), designer (N = 1), other (N = 4)	Job coach characteristics and characteristics of supported employees (age, gender, education, experience, training, caseload, primary disability and time at current job)	Evaluation of the supported employee, additional training, supervision, personal relations and responsiveness of the job coach to employers’ needs were important for job retention	Questionnaire for employers and job-coaches

## Results

### Description of Studies

After a search in five databases yielding with 1932 articles, 33 articles underwent methodological quality assessment in the fourth phase. After careful and independent screening, 26 articles remained. Most frequently occurring criteria for exclusion of a paper based on the quality criteria of Buckley et al. were the absence of: ethical issues (n = 6), reproducibility (n = 6), completeness of data (n = 6), data collection methods (n = 6) and information of study subjects (n = 5).

Of the 26 included articles, 18 were from the USA and the remainder were from Hong Kong (n = 2), Canada (n = 2), Spain (n = 1), Japan (n = 1), Australia (n = 1), and New Zealand (n = 1). Of all articles, 19 were based on qualitative research and seven on quantitative research. Three articles were based on longitudinal data with follow-up periods ranging from 18 months to 4 years. In 15 studies the sample size was <50 persons and, of these seven had a sample size of <10 persons. Many studies either did not use the definition of the AAIDD, or lacked a clear definition of the level of the ID. One article focused on people with moderate ID (IQ 35–50), one focused on people with severe ID (IQ 20–35) and four focused on people with mild ID (IQ 50–75). Six articles were based on a combination of types of ID. All other articles mentioned ID without further specifying the intellectual level or individual functioning.

The study population of the included studies consisted of employers (six articles), employees (15 articles), employees with ID and co-workers (three articles) and a combination of employers, employees with ID and job coaches (two articles). Although employees with ID were study subject of 15 studies they were interviewed in four studies. In most studies employers, researchers and supervisors gave information about the work done by individuals with ID.

The jobs performed by people with ID differed greatly, although many belonged to unskilled or entry level jobs with an emphasis on jobs in shops, offices, or in the food (industry), janitorial and manufacturing areas. Competitive employment had many features in the included studies. This ranged from clustered placement at workplaces also called enclaved placement or community-based jobs, to individualized work settings [37]. The work settings and work content differed to a great extent referring to the total hours of work per week, earnings, type of support at the workplace and the size of organizations involved. Of the selected studies, eight used criteria for relatively short working periods as a basis for participation in the studies, ranging from two to (at least) 6 months. In three studies the participants had worked for (at least) 1–20 years. Eight studies provided no information on how long their participants had been working. Because of these different outcomes, variables, work settings and lack of empirical support no generalizations on effective methods that supports work participation of people with ID can be given.

### Outcome of Data Extraction into Four Categories

Based on data extraction, four themes/categories with work environment-related factors that could influence work participation were distinguished (see Table 2). Although aspects of more than one category were sometimes mentioned in the studies, the categories were classified based on the main topics described in the study. Outcomes were classified in the following categories: (1) employers’ decisions and opinions; (2) job content; requirement and performance; (3) interaction and workplace culture; and (4) support from job coaches.

1. Employers’ decisions and opinions

Five studies focused on the perspective of employers’. Safety, productivity, attendance, availability of supportive services, no behavioral problems and punctuality were important aspects in the decision-making process to hire people with ID [38, 39]. Not many employers reported having hiring experiences with employees with ID. Those having experience with hiring employees with ID was found to be stimulating for employers for doing that again [40]. Experienced employers expressed more favorable attitudes, perceived more advantages and fewer disadvantages to these employees than did inexperienced employers [41]. The most positive views on work participation for people with ID were found among businesses in the USA with >200 employees [40].

Social altruism was mentioned by employers as an incentive work environment-related factor in which personal responsibility for breaking stereotypes and eliminating bias in the workplace were important aspects [41]. Another study [42] reported the high motivation level of employees with ID and the importance of not compromising the expectations of employers and employees with ID to arrange successful matches and good performances of these employees.

2. Job content, requirement and performance

Eight studies focused on promoting factors for job content, requirement and performance of employees with ID. Activities to enhance performance of employers were for example: training for performing job tasks with the use of specific words, a model to support working with self-selected goals and how to reach them, and a model used for clarifying support needs of employees with ID [43–45]. Referring to job content, clerical, custodial and manufacturing work were among the 11 distinguished tasks most often performed by employees with ID [46].

Describing how job matches were made and paying attention to job development were found important factors for job performance and maintaining work [46, 47]. Another study stressed the need of reduced job demands and support to acquire a higher quality of work life [48]. Employees with ID felt that they were sometimes confronted with misconceptions about their possibilities [49]. Training to improve work performance and self-advocacy were appointed as relevant factors for obtaining a job and creating an atmosphere where employees with ID felt supported [49]. Individually placed persons in a work setting resulted in larger wage gains than did the enclave placement approach. The approach that appeared to result in higher wages was providing subsidies to employers [37].

3. Interaction and workplace culture

Workplace culture is a broad concept in which interaction, integration, social support, and participation were mentioned as relevant aspects for obtaining and maintaining work. Therefore, these themes were combined in the category ‘interaction and workplace culture’. Eight articles focused on these themes. Specific social opportunities, personnel and teambuilding, management style, attention for diversity at the workplace, flexibility, structure, supervision, and job creation were mentioned as important factors for employability of employees with ID in regular workplaces [5, 50, 51]. Employees with ID added to the workplace culture in a positive way through the dedication to their work. They were mostly proud of their jobs [5].

Another main theme was the importance of interaction in the workplace as value for being accepted [31, 52, 53]. Social support on different levels, from co-workers, bosses, job coaches and families was found to be relevant for maintaining jobs for people with ID [54]. In addition the importance of parents as a form of social capital that had to be used in supporting community employment was emphasized [52].

Existing disability and social assistance programs could however be seen as obstructing factors for competitive employment. A paid job can be viewed as a ‘treat’ to existing benefits, because benefits will stop when paid work is found without security of job retention [52].

4. Support by job coaches

Support by job coaches was a topic of research in five studies. Job coaches fulfilled relevant roles for entrance in the workforce and support in the workplace itself, by specific training for job readiness and on-the-job training to improve the work performance, productivity, safety and employability of these employees [55, 56]. Receiving support from job coaches on the work place was seen as a relevant factor in maintaining a job and seen as a form of instrumental support [55]. The availability of job coaches was a facilitating factor for employers in hiring employees with ID if they were responsive to the employer’s needs [57]. Two articles stressed the role of the job coach as an important factor for employers in their decision-making process to recruit employees with ID [38, 40].

## Discussion

This review examined which work environment-related factors contribute to obtaining or maintaining work in competitive employment for employees with ID. In total 26 articles were identified after a systematic review of relevant literature published over the past 20 years. Our extensive investigation revealed that this field, concerning employees with ID in a regular work setting, is a relatively unexplored area of research. Few data were available on this specific group of employees in relation to work, despite ongoing legislation and promotion of supported employment. And although the definition of the AAIDD was used as criterion for ID a classification into moderate, mild and severe ID was most often used in the studies. It was nevertheless not reported by all studies. Based on these findings it was difficult to make generalizations directed at the level of ID of the employees.

The findings of the results of this review provide insight into some important aspects of work environment-related factors that could influence work participation. Four categories were distinguished: (1) employers’ decisions and opinions; (2) job content, requirement and performance; (3) interaction and workplace culture; and (4) support from job coaches. Knowledge on and activities related to these categories were found to be important to facilitate participation in regular paid employment of employees with ID but more evidence based research is required on these themes. Striking in the studies is the limited presence of both employers and employees with ID.

Few studies focused on the employers, although they are relevant decision makers in creating work places for people with ID. Positive and negative public perceptions are important aspects to pay attention to in building effective programs for work participation of employees with ID and in developing models of good practices [4]. Employers’ views on people with ID can be divided into three different tendencies: negative stereotyping, disengagement, and positive experiences [21]. Employers who had experience with employees with ID had a more positive attitude toward these employees compared with employers without such experiences [40]. Employers with these positive experiences can be helpful as relevant stakeholders in supporting work participation. Walgreens was mentioned as a leader in business in hiring persons with significant disabilities and an example that more employers are willing to follow [57]. Enhanced understanding of disability is supportive in creating disability awareness in the workplace. That can be seen as a relevant factor in supporting work participation of employees with ID [4].

Expanding and improving relationships between employers and support services can create a greater focus on work participation of employees with ID. Some studies emphasized that the availability of support services, responsiveness to employers’ needs of job coaches and additional preparation and training were relevant factors in affecting employers’ decisions [38, 39, 57]. More attention could be paid to these factors. The connection between employers and support agencies in finding employees with ID was subject in only one study. This study showed that the job coach usually made the connection with employers [42]. Another aspect that could be relevant in supporting work participation are subsidies for employers concerning this specific group of employees although this was not an issue in many studies [14, 37].

Support of job coaches was found an effective service for employees with ID [5, 56]. How this support is organized and the contribution of job coaches in influencing workplace operations and employers’ decisions are important aspects for support at the workplace [56]. Although training on the job and use of specific instruments or models were mentioned as important factors to support work participation for people with ID most research was based on small sample sizes or only one empirical study [43, 45]. Based on these findings it is difficult to make generalizations that could be useful for more knowledge of effective training programs for training on the job and problem solving methods.

Different studies confirmed the importance of support systems and assistance at the workplace as relevant factors for integration [16, 31]. Integration in a workplace is a complex process that asks for understanding of the perspectives of the different stakeholders and the interdependency between different kinds of support [51, 54]. Sources of support in the workplace that were described were support by co-workers, managers, job coaches and even families [31, 58]. Related to the culture of an organization commitment and attention for flexibility, leadership and diversity were important in obtaining and maintaining work for people with ID [5, 52]. Attention needs to be paid to interrelations between the different support systems. For example how can support of job coaches, co-workers and managers reinforce each other in creating more work participation of employees with ID? In the field of different interrelations the role of families looks under-represented. Only two of the included studies paid attention to families as relevant support partners [52, 54].

Although some studies have indicated that employees with ID had specific skills other studies described that employees with ID were confronted with misconceptions about their capacities or discrimination [4, 42, 49]. Underestimation and negative attitudes of colleagues seemed a relevant issue that was also described in a review concerning disabled people as a more general group without specific attention for employees with ID [59]. Understanding factors that create stigma or discrimination needs more attention in promoting the employment of employees with ID in the general labor market.

Employees with ID seem overlooked in many studies what was confirmed in a review of inclusion of employees with ID in workplaces [33]. In the majority of the 26 studies of this review the employees’ perspective was not included. Only four studies interviewed employees with ID. Overall can be said that their meaning is underexposed. Data were obtained by supervisors or job coaches who filled in questionnaires, observations by researchers or from databases of providers. The opinion of employees with ID themselves could be more elaborated. Self-advocacy of employees with ID supported by job coaches or families in promoting dedication to their work in combination with disability education for employers’, could be helpful in generating ID awareness by employers and reducing underestimation of capacities of workers with ID. This can possibly contribute to empowerment by employees with ID in promoting work participation [49, 60].

For employees with ID existing benefit systems can be obstructive in relation to work participation because these systems will stop when they start working in the regular employment [46, 52]. More differentiation between benefit systems for employees with ID and income from work can be supportive in work participation for this specific group because they still are vulnerable in bad economic times.

Inclusive workplaces are those places where not only employees with ID benefit from attention for specific support but even all others involved in the same workplace [33]. Aspects as management style, flexibility, sense of belonging, supervision and teambuilding in different workplaces were mentioned as relevant factors. These aspects were described in general terms and more specific knowledge in the meaning of these factors, their interrelations and how they are usable for distinguishable work cultures lacked in the included studies.

In work participation aimed at different groups of disabled employees the person, the job and the work environment were seen as important factors in need of examination with specific attention to support and social relations [16]. In the field of work participation of employees with ID results from research confirm that evidence for factors that are associated with sustainable work participation are limited [1]. Specific attention for environmental factors related to work participation in research for employees with psychiatric problems is even lacking although more research is done in this field compared to the field of ID [22, 61, 62].

### Limitations of this Review

This research has some limitations that need to be acknowledged. Most of the selected studies in our review were conducted in the USA. That means that our results may not be applicable to other countries because local conditions can be different. Thereby our search strategy was limited to the English language within a specific time period. That means that studies in other languages relevant to the scope of this review may have been overlooked even as studies conducted before or after the specific time period.

A second limitation has to do with the lack of controlled outcome studies in this area. In studies addressing the different topics concerning work environment-related factors in obtaining and maintaining work the outcomes differed and were not comparable. Almost no controlled effect studies has been done for successful interventions although different studies stress the importance of the job coaches [55, 63]. More comparable data and factors are required to understand the results and use them for building knowledge on this issue.

### Implications for Future Research

More studies are required that focus on inclusive work settings where employees with ID are welcome as valued employees. Aspects of employers’ opinions and views about employees with ID by colleagues and general public need to be investigated with specific attention to stigma and discrimination. In creating successful inclusive work settings relevant aspects of the work environment, the work culture, job content, leadership, size of organizations, instruments and training programs need to be investigated more precisely. Additional longitudinal research in comparable settings is required in a broader context than the USA.

Effective support mechanisms and understanding of reciprocal interdependency between different sources of support for employees with ID should be investigated as relevant factors of a work environment. In this research employees with ID, employers, job coaches and family members are equally important. They all fulfill different sources of support that can be helpful in improving employment outcomes for people with ID. Opinions and values of all these different stakeholders need to be taken into account with specific attention for inclusive and discriminating issues. This requires multi-level research in what is needed to create transitions in the labor market for opening this market for employees with ID. Work environment-related factors including policies and funding mechanisms need to be investigated.

Future research should evaluate the methods used by job coaches for employees with ID to influence and support work participation. In this research workplace operations, relations between job coaches and employers, the use of specific training models or—instruments, supporting employers’ decision processes and facilitating entrance to work seem important issues. The research will be helpful in professionalizing the function of the job coach. This is important in providing a contribution to more evidence-based practices in this field.

To support the issue of work participation of employees with ID more research could be helpful to understand their wishes and opinions and pay attention to self-advocacy as an empowering model in work participation. Specific attention needs to be paid to the role of existing benefit systems and how they empower or obstruct work participation of employees with ID.

## Conclusion

The field of work environment-related factors in obtaining and maintaining work in competitive employment for employees with ID is a relatively unexplored area. Relevant issues are employers, the job content and performance, interaction and workplace culture and job coaches. More research is needed on these issues to promote work participation with specific attention for employers and employees with ID.

## Electronic supplementary material

Supplementary material 1 (DOCX 12 kb)

Supplementary material 2 (DOCX 14 kb)
