# Supplementary material for: Work Environment-Related Factors in Obtaining and Maintaining Work in a Competitive Employment Setting for Employees with Intellectual Disabilities: A Systematic Review
Source: J Occup Rehabil. 2015 Jun 26;26:56–69. doi: 10.1007/s10926-015-9586-1 (PMC4749651; doi:10.1007/s10926-015-9586-1)
Supplement: Supplementary file 1 — Supplementary material 1 (DOCX 12 kb) [file 10926_2015_9586_MOESM1_ESM.docx]

**Appendix 1**

| **Scheme with relevant search terms** | |
| --- | --- |
| **Definitions used for search in different databases** | |
| **POPULATION** | **OUTCOME** |
| **People with intellectual disability** | **Work** |
| Intellectual disability  Learning disability  Mental retardation  Developmental disability  Intellectual development disorder  Intellectual impairment | (Open)Employment  (Paid)Work  Job  Employability  Labour/labor participation  Competitive employment  Supported employment  Remunerated employment  Occupation  Vocational rehabilitation |
