# Supplementary material for: Work Environment-Related Factors in Obtaining and Maintaining Work in a Competitive Employment Setting for Employees with Intellectual Disabilities: A Systematic Review
Source: J Occup Rehabil. 2015 Jun 26;26:56–69. doi: 10.1007/s10926-015-9586-1 (PMC4749651; doi:10.1007/s10926-015-9586-1)
Supplement: Supplementary file 2 — Supplementary material 2 (DOCX 14 kb) [file 10926_2015_9586_MOESM2_ESM.docx]

**Appendix 2
Used ‘search-strings’ in five databases**

**Database Web of Science**

Title=(mental retardation OR mental deficiency OR developmental disabil* OR intellectual disabil* OR intellectual developmental disorder*) AND Title=(employment OR supported employment OR vocational rehabilitation OR occupation* OR work OR job OR labor)

Timespan=1993-2013. Databases=SCI-EXPANDED, SSCI, A&HCI, CPCI-S, CPCI-SSH.

|  |  |
| --- | --- |

*Results: 344 hits*

**Database Embase**

#2: 'employment'/exp/mj OR 'vocational rehabilitation'/exp/mj OR 'occupation'/exp/mj OR employement:ti OR 'supported employment':ti OR 'vocational rehabilitation':ti OR work:ti OR job:ti OR employability:ti OR 'competitve employment':ti OR 'competitve employment':ab OR 'paid work':ti OR 'paid work':ab OR 'labor participation':ti OR 'labor participation':ab OR occupation:ti OR occupation:ab AND ([adolescent]/lim OR [adult]/lim) AND [humans]/lim AND [english]/lim AND [embase]/lim AND [1-1-1993]/sd NOT [31-12-2013]/sd: [15,738](http://www.embase.com.proxy.library.uu.nl/search/results?viewsearch=4)

#1

'mental deficiency'/exp/mj OR 'mental deficiency':ti OR 'mental deficiency':ab OR 'mental retardation':ti OR 'mental retardation':ab OR 'developmental disorder'/exp/mj OR 'intellectual disabilities':ti OR 'intellectual disabilities':ab OR 'intellectual developmental disorder':ti OR 'intellectual impairment':ti AND ([adolescent]/lim OR [adult]/lim) AND [humans]/lim AND [english]/lim AND [embase]/lim AND [1-1-1993]/sd NOT [31-12-2013]/sd: [13,409](http://www.embase.com.proxy.library.uu.nl/search/results?viewsearch=3)

#3 : #1 AND #2

*Results: 112 hits*

**Database CINAHL**

S1
MM Employment OR MH "Employment of Disabled+" OR MM "Rehabilitation, Vocational" OR MM Work OR MM "Occupations and Professions"

S2
TI employment OR AB employment OR TI supported employment OR AB supported employment OR TI work OR TI job OR AB job OR TI occupation* OR AB occupation* OR TI employability OR AB employability OR TI labour participation OR AB labour participation OR TI competitive employment OR AB competitive employment OR TI remunerated employment OR AB remunerated employment OR TI paid work OR AB paid work OR TI open employment OR AB open employment

S3

S1 OR S2

S4
MM "Mental Retardation+" OR MM "Developmental Disabilities" OR MM "Learning Disorders"

S5
TI mental retardation OR AB mental retardation OR TI intellectual disabilit* OR AB intellectual disabilit* OR TI developmental disabilit* OR AB developmental disabilit* OR TI intellectual developmental disorder* OR AB intellectual developmental disorder* OR TI intellectual impairment* OR AB intellectual impairment* OR TI mental deficiency OR AB mental deficiency

S6

S4 OR S5

S7

S3 AND S6

S8
TI child* OR AB child* OR TI infant* OR AB infant*

S9
S7 NOT S8; limiters: Published Date from: 19930101-20131231; Human; Language: English; Age Groups: Adolescent: 13-18 years, Adult: 19-44 years, Middle Aged: 45-64 years

*Results: 190 hits*

**Database PsycINFO**

S1
MM Employment Status OR TI employment OR AB employment OR MM Supported Employment OR MM Vocational Rehabilitation OR TI work* OR TI job OR AB job OR MM Occupations OR TI employability OR TI labor participation OR TI labour participation OR TI competitive employment OR AB competitive employment OR TI remunerated employment OR AB remunerated employment OR TI paid work OR AB paid work OR TI open employment OR AB open employment OR TI occupation* OR AB occupation*

S2
MM Intellectual Development Disorder OR MM Developmental Disabilities OR TI developmental disabilit* OR AB developmental disabilit* OR TI intellectual developmental disorder* OR AB intellectual developmental disorder* OR TI mental retardation OR AB mental retardation OR TI intellectual impairment* OR AB intellectual impairment* OR MM Learning Disabilities

S3
child* OR infant*

S4
(S1 AND S2) NOT S3. Limiters: Published Date from: 19930101-20131231; English; Age Groups: Adolescence (13-17 yrs), Adulthood (18 yrs & older), Young Adulthood (18-29 yrs), Thirties (30-39 yrs), Middle Age (40-64 yrs)

*Results: 787 hits*

**Database PubMed**(employment[majr] OR employment[tiab] OR "employment, supported"[majr] OR "rehabilitation, vocational"[majr] OR work[majr] OR job[tiab] OR employability[tiab] OR labor participation[tiab] OR labour participation[tiab] OR competitive employment[tiab] OR (remunerated[tiab] AND employment[tiab]) OR paid work[tiab] OR open employment[tiab] OR occupation*[tiab] OR occupations[majr]) AND (intellectual disability[majr] OR intellectual disabilit*[tiab] OR developmental disabilities[majr] OR developmental disabilit*[tiab] OR intellectual development disorder*[tiab] OR intellectual impairment*[tiab] OR mental retardation[tiab] OR mental deficiency[tiab] OR learning disabilit*[tiab]) NOT (Child[mh] OR infant[mh] OR child*[ti] OR infant*[ti]) Filters: Publication date from 1993/01/01 to 2013/12/31; English

*Results: 499 hits*

**Total results out of all databases: 1932 hits.**
